# A Weld Joint Type Identification Method for Visual Sensor Based on Image Features and SVM

**DOI:** 10.3390/s20020471

**Published:** 2020-01-14

**Authors:** Jiang Zeng, Guang-Zhong Cao, Ye-Ping Peng, Su-Dan Huang

**Affiliations:** Shenzhen Key Laboratory of Electromagnetic Control, College of Mechatronics and Control Engineering, Shenzhen University, Shenzhen 518060, China; zengjiang525@163.com (J.Z.); yeping.peng@szu.edu.cn (Y.-P.P.); hsdsudan@gmail.com (S.-D.H.)

**Keywords:** weld joint type identification, image feature extraction, visual sensor, support vector machine (SVM)

## Abstract

In the field of welding robotics, visual sensors, which are mainly composed of a camera and a laser, have proven to be promising devices because of their high precision, good stability, and high safety factor. In real welding environments, there are various kinds of weld joints due to the diversity of the workpieces. The location algorithms for different weld joint types are different, and the welding parameters applied in welding are also different. It is very inefficient to manually change the image processing algorithm and welding parameters according to the weld joint type before each welding task. Therefore, it will greatly improve the efficiency and automation of the welding system if a visual sensor can automatically identify the weld joint before welding. However, there are few studies regarding these problems and the accuracy and applicability of existing methods are not strong. Therefore, a weld joint identification method for visual sensor based on image features and support vector machine (SVM) is proposed in this paper. The deformation of laser around a weld joint is taken as recognition information. Two kinds of features are extracted as feature vectors to enrich the identification information. Subsequently, based on the extracted feature vectors, the optimal SVM model for weld joint type identification is established. A comparative study of proposed and conventional strategies for weld joint identification is carried out via a contrast experiment and a robustness testing experiment. The experimental results show that the identification accuracy rate achieves 98.4%. The validity and robustness of the proposed method are verified.

## 1. Introduction

Robotic welding technology is an important indicator on which to measure the technical development of the welding industry in today’s highly developed environment. Currently, the two most common operating modes for welding robots, namely, the teaching mode and the off-line programming mode, do not depend on sensor measurements during welding; the welding trajectories are set in advance by workers, and the robot moves in accordance with the desired trajectory. These two modes are suitable for use in a standardized, modular, strictly coordinated welding system [1,2,3,4]. However, in actual welding operations, the welding environment might not be static. Therefore, these two modes do not offer sufficient flexibility and robustness to handle such a complex and dynamic welding environment. The teaching mode and off-line programming mode require a lot of time to teach and preprogram each time the workpiece is replaced; therefore, with the rapid improvement in sensor technology and integration technology, intelligent welding robots that can overcome the difficulties that are encountered when operating welding robots in the teaching and off-line programming modes are emerging. An intelligent welding robot uses external sensors to perceive its environment and detect the weld position. In the past, arc and contact sensors were two of the most widely applied types of sensors in robot sensing models [5,6]. Visual sensing is widely used presently in the field of robotic welding because of its high accuracy and non-contact nature [7,8,9]. Furthermore, it could obtain abundant information regarding the welding environment. Currently, most visual sensors are composed of a camera, an auxiliary laser, a filter, and a partition. In general, the auxiliary laser is a line laser. Research that is related to visual sensors typically focuses on image preprocessing [10,11,12], weld joint contour extraction [13,14,15], and weld feature point extraction [16,17,18].

The recognition of the weld joint is the most important part of weld visual sensors. In real industrial environments, many types of weld joints are encountered, including fillet weld joints, butt weld joints, and v-type weld joints, due to the diversity of the workpieces. Many scholars have proposed corresponding single weld joint recognition methods for locating the position of the weld joint [10,11,12,13,14,15,16,17,18]. For example, Fan et al. considered feature extraction for butt weld joints [17], Zou et al. considered feature extraction for the recognition of lap weld joints [18], and Fang considered feature extraction for fillet weld joints [19]. These methods need to recognize only one kind of weld joint due to the single weld joint environment. However, in some welding environments, such as bridge structure welding environments, there might be many kinds of weld joint types. Different weld joints have different welding currents, welding voltages, welding torch swing methods, and welding speeds. Therefore, the ability to recognize multiple types of weld joints is necessary in a real welding environment. In recent years, some researchers have investigated multi-type weld joint recognition. Li et al. used the spatial composition relationship of the line segment elements and the junctions of weld joints for weld joint classification [20]. First, the relationship between the line segment elements and the junctions is defined; then, weld joint recognition is performed in accordance with this characteristic composition relationship. This method is feasible, but it has difficulty in recognizing different weld joints with similar compositional characteristics. Qian et al. proposed a two-step method for multi-type weld joint recognition [21]. First, the position of the laser curve is found and then the weld type is recognized. This method identifies the weld joint type by considering the correlations among feature points However, while using this method, a series of experiments must be performed to choose threshold values to distinguish different weld joints, which is complex and impractical. Li et al. proposed a method that was based on the Hausdorff distance and template matching to recognize different weld joint types [22]; its computational cost is 1.17s and it lacks adaptability. Fan et al. proposed a method for establishing a support vector machine (SVM) model by using the distances from the weld joint ends to the bottom of the weld to form the feature vector [23]; it has a better recognition accuracy and less computational cost than other methods, possibly because it uses SVM to build the recognition model.

SVM is a machine learning algorithm that is widely used for classification. This algorithm is very suitable for problems that involve small sample sizes and it has a relatively low computational cost. Additionally, the SVM approach is also suitable for solving nonlinear, high-dimensional problems because its use of a kernel function. SVM was applied in [23]. However, for two similar weld joints, the recognition results are poor because the feature vector only has one kind of feature and, thus, does not have enough recognition information. Therefore, the development of a weld joint recognition system that has high recognition accuracy and low computational cost and is suitable for multiple types of weld joints is necessary.

In this paper, a weld joint type identification method for visual sensor based on image features and SVM is proposed. Two different types of features are extracted from the weld joint image to improve the recognitions accuracy. SVM is used to build a model for weld joint type recognition to reduce the computational cost and improve the accuracy of recognition, and five-fold cross-validation is employed to find the optimal parameters of the SVM model.

The main contribution of this paper is three-fold, as follows:

(1) A weld joint type identification method that is based on the deformation information of a laser curve in the image is proposed to improve the welding system adaptability and automation degree.
(2) A weld joint type identification model that is based on an optimal SVM is constructed to identify various kinds of weld joints.
(3) A comprehensive study on the feature extraction, recognition, experimental comparison, and discussion of weld joint images is performed to handle the weld joint recognition task of the arc welding systems.

The remaining sections of this paper are organized, as follows. Section 2 introduces the visual tracking sensor used in this paper and the feature analysis of various welding joint images. In Section 3, the weld joint image feature extraction method used in this paper is proposed. In Section 4, an SVM-based weld joint recognition model is built, and the weld joint type recognition method proposed in this paper is introduced. Section 5 discusses the experimental. Finally, the conclusions of this paper are provided.

## 2. Visual Sensor and Image Features Analysis of Weld Joints

### 2.1. Visual Sensor

**Figure 1:**
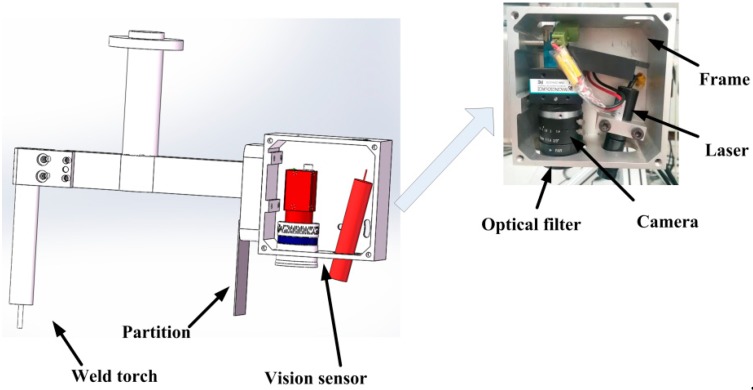
(**Left**) weld torch and visual sensor model; (**Right**) visual sensor.

All of the weld joint images that were considered in this paper were obtained while using our independently developed visual sensor, as shown in Figure 1.

The visual sensor is equipped with a complementary metal-oxide-semiconductor (CMOS) camera, the parameters of which are shown in Table 1. The main function of the laser is to project a laser curve on the workpiece for features extraction and three-dimensional (3D) reconstruction. The partition is designed to isolate splash to reduce the noise in the image. The laser wavelength that was used in this paper as 650 nm and the laser power was 30 mW. The adjustable width of the fringes ranges from 0.5 mm to 2.5 mm. The penetration of filters is 80%; the filters filter splash and light. The focus length is 12 mm. The distance between camera and welding torch is 200 mm. In a real welding environment, the posture of the visual sensor will be adjusted in real time to keep it perpendicular with the weld joint, so that the laser curve is vertical in the weld joint image, that is, the visual sensor is oriented the same way relative the path, even for curved paths.

**Figure 2:**
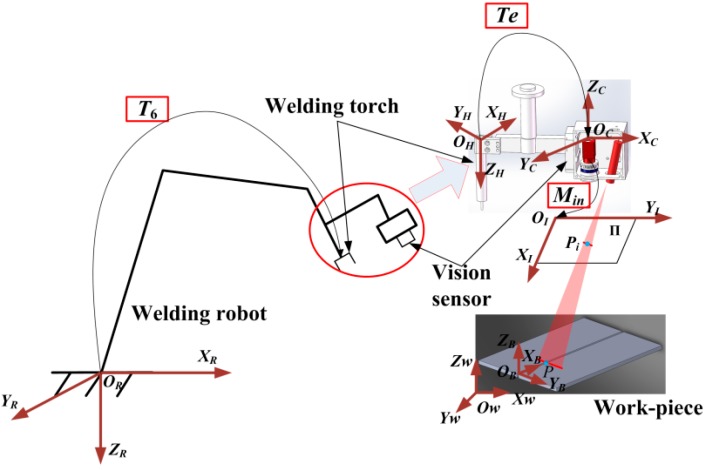
Coordinate transformation model.

The visual sensor is used to capture the two-dimensional coordinates of weld feature point in the pixel coordinate system, to map it to robot base coordinate system through a series of transformations matrix. Figure 2 shows the coordinate transformation model.

*O_W_X_W_Y_W_Z_W_* is world coordinate system, *O_I_X_I_Y_I_* is pixel coordinate system, *O_C_X_C_Y_C_Z_C_* is camera coordinate system, *O_H_X_H_Y_H_Z_H_* is robot welding torch coordinate system, *O_R_X_R_Y_R_Z_R_* is robot base coordinate system. *O_B_X_B_Y_B_Z_B_* is workpiece coordinate system, *T*_e_ is hand-eye matrix relating *O_C_X_C_Y_C_Z_C_* to *O_H_X_H_Y_H_Z_H_*, as obtained by hand-eye calibration [24], *T*_6_ is transformation matrix between *O_H_X_H_Y_H_Z_H_* and *O_R_X_R_Y_R_Z_R_*, as obtained by the robot controller, ***M****_in_* is the transformation matrix between *O_C_X_C_Y_C_Z_C_* and *O_I_X_I_Y_I_*, obtained via camera calibration [25], Π_1_ is the imaging plane, *P* is one of the projection points of the laser on the surface of the welding work-piece, and *P_i_* (*X_I_*, *Y_I_*) is the image point that corresponds to *P* in the pixel coordinate system. The laser plane can be represented by *Ax* + *By* + *Cz* = 1. It can be calculated by laser plane calibration [26]. Therefore, the 3D camera coordinate of *P* (*X_C_*, *Y_C_*, *Z_C_*) can be represented by *P_i_*, camera intrinsic parameters and laser plane, as follows

(1) $$\{\begin{array}{c} X_{c}=\frac{k_{y}(X_{I}-X_{I0})}{Ak_{y}(X_{I}-X_{I0})+Bk_{x}(Y_{I}-Y_{I0})+Ck_{x}k_{y}}\\ Y_{c}=\frac{k_{x}(Y_{I}-Y_{I0})}{Ak_{y}(X_{I}-X_{I0})+Bk_{x}(Y_{I}-Y_{I0})+Ck_{x}k_{y}}\\ Z_{c}=\frac{k_{x}k_{y}}{Ak_{y}(X_{I}-X_{I0})+Bk_{x}(Y_{I}-Y_{I0})+Ck_{x}k_{y}} \end{array}$$

where *X_I_*, *Y_I_* is coordinate of point *P_i_* in pixel coordinate system, *Xc*, *Yc*, *Zc* is the coordinate of point *P* in camera coordinate system, the parameters *k_x_* = *f*·*m_x_*, *k_y_* = *f*·*m_y_* represents the focus length in terms of pixels, where *m_x_* and *m_y_* are the scale factors that relate pixels to distance and *f* is the focal length in terms of distance. *X_I_*_0_ and *Y_I_*_0_ represent the principal point, which would be ideally in the center of the image.

Finally, we can establish the mapping matrix between a point in the image coordinate system and the corresponding point in the robot base coordinate system through the coordinate transformation model, that is:

(2) $$\left[\begin{array}{c} X_{R}\\ Y_{R}\\ Z_{R}\\ 1 \end{array}\right]=T_{6}T_{e}\left[\begin{array}{c} X_{C}\\ Y_{C}\\ Z_{C}\\ 1 \end{array}\right]$$

### 2.2. Image Features Analysis of Weld Joints

**Figure 3:**
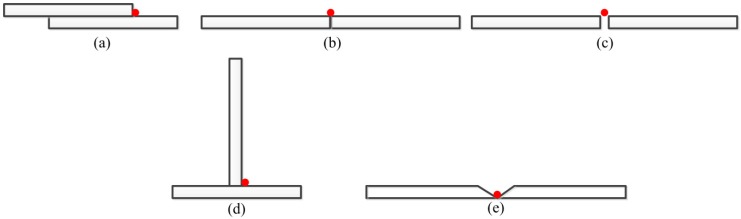
Weld joint types: (**a**) lap weld joint, (**b**) butt weld joint, (**c**) splice weld joint, (**d**) fillet weld joint, and (**e**) v-type weld joint.

In a real welding environment, based on the groove shape, the groove size, and the interval between and relative positioning of the two welding work-pieces, a weld joint can be classified as one of four types, i.e., lap weld joint, butt weld joint, fillet weld joint, and v-type weld joint, as shown in Figure 3. It should be noted that Figure 3b,c both show a butt weld joint; therefore, we use the term “splice weld joint” in Figure 3c to differentiate them.

**Figure 4:**
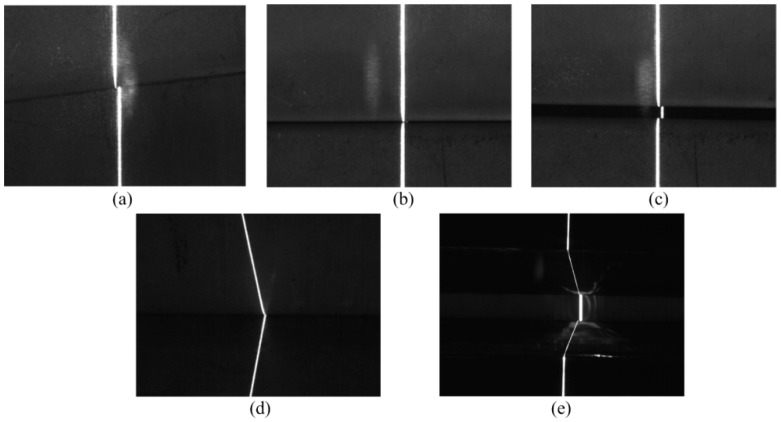
Weld joint images with laser stripe: (**a**) lap weld joint, (**b**) butt weld joint, (**c**) splice weld joint, (**d**) fillet weld joint, and (**e**) v-type weld joint.

**Figure 5:**
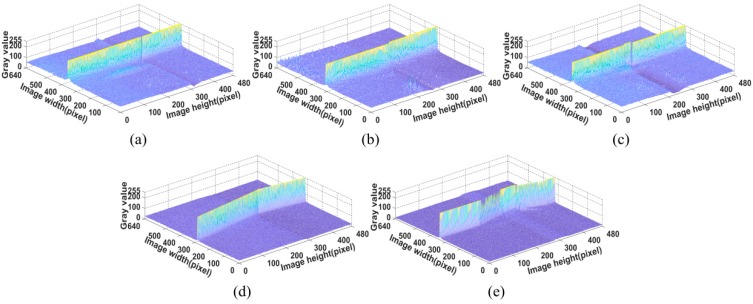
Grey value intensity of weld joint: (**a**) lap weld joint, (**b**) butt weld joint, (**c**) splice weld joint, (**d**) fillet weld joint, and (**e**) v-type weld joint.

Figure 4 shows the weld joint images that were obtained under laser projection while using our visual sensor. The size of the image is 640 × 480 pixels and the thickness of laser curve in the image is approximately 5–8 pixels in the weld joint image. The size of v-type joint work-piece samples is 800 mm × 150 mm and the others are 600 mm × 150 mm. Figure 5 shows the corresponding grey value intensity distributions of Figure 4.

An analysis of the weld joint images and the grey value intensity distribution diagrams reveals that the various weld images exhibit the following characteristics:

(1) The distinguishing features of a weld joint image are only related to the laser stripe in the image. It is difficult to recognize a weld joint that is based on another part of the image, except for the part near the laser stripe.
(2) The laser stripe in the weld joint image has the characteristics of discontinuity, grey value change, and deformation. For different weld joints types, the degrees of deformation and the change of grey value of the laser stripe in the image also differ.

According to the above analysis, the abrupt differences of laser stripe at weld joints in image can be used as features to describe and, thus, recognize different weld joints.

## 3. Image Features Extraction of Weld Joints

**Figure 6:**
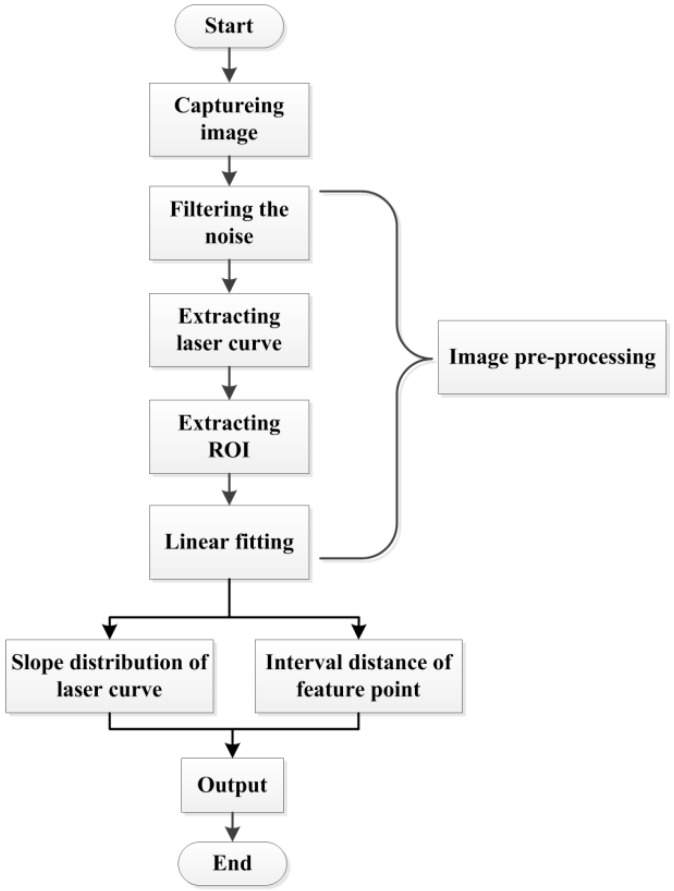
Features extraction process of a weld joint image.

Image features determines the recognize accuracy and applicability of a weld joint recognition system. In this paper, we take full advantage of the laser stripe deformation information in the image as the features for identifying the weld joint type. Figure 6 outlines the extraction process.

### 3.1. Weld Joint Image Preprocessing

The laser curve in the image must be extracted to extract the feature vector. Therefore, weld joint image preprocess include noise processing, laser curve extraction, region of interest (ROI) extraction, and laser curve fitting.

#### 3.1.1. Noise Processing and Laser Curve Extraction

**Figure 7:**
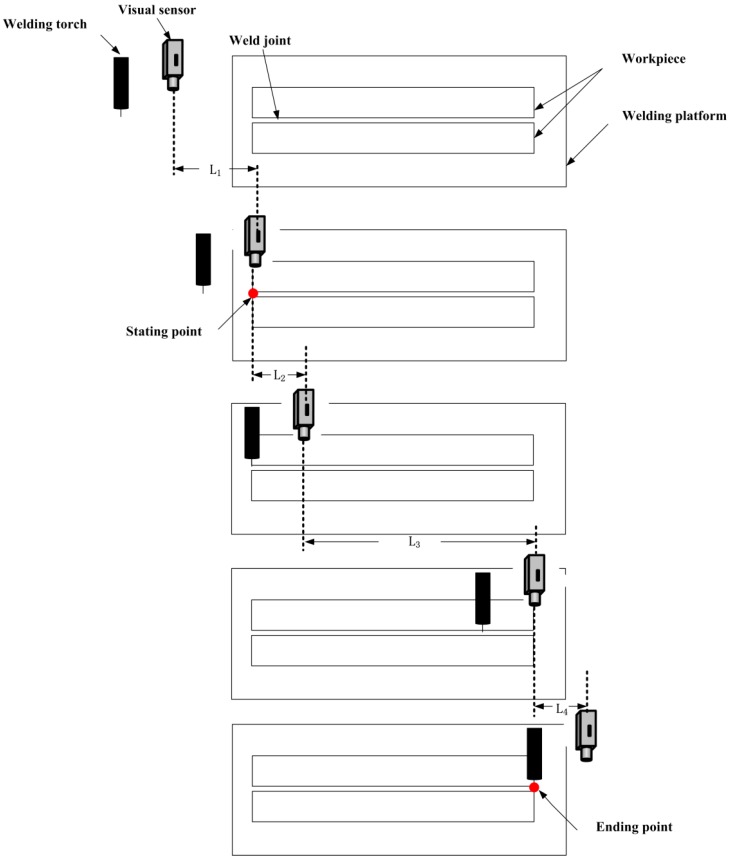
The relative position of the welding torch, visual sensor and the workpiece.

As shown in Figure 7, regarding the relative position of the visual sensor, the welding torch and the workpiece, the welding process is divided into four stages:

L1: The robot start moving–The visual sensor arrive the weld starting point

Set the scanning path before the welding operation and use the visual sensor to find the starting point of the weld based on the imaging difference between the weld joint image and background image. At this stage, the visual sensor has not captured the weld joint image and the welding torch does not start welding.

L2: The visual sensor arrive the weld starting point–The welding torch arrive the weld starting point

Determine whether the visual sensor has captured the weld joint image. The visual sensor will identify the weld joint types when the weld joint image is captured. At this stage, the welding torch does not arrive the starting point of the weld joint and does not perform welding. Therefore, the obtained image is without splash and other interference.

L3: The welding torch arrive the weld starting point–The visual sensor arrive the weld ending point.

When the welding torch arrive the weld starting point, the welding torch starts welding. The image processing method of weld joint location and relevant welding parameters used in this stage are based on the identification result in L2. In this stage, there is a large amount of splash interference in the image that was captured by the camera.

L4: The visual sensor arrive the weld ending point–The welding torch arrive the weld ending point

When the visual sensor captures the welding ending point, the welding scanning is completed. Once the welding torch reaches the welding ending point, welding is completed.

**Figure 8:**
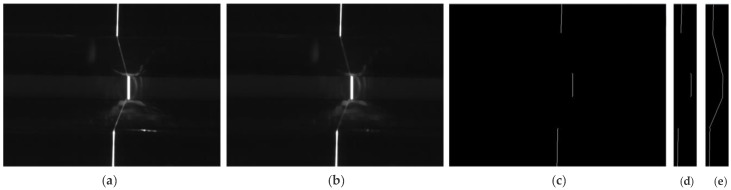
(**a**,**b**) Image noise processing result of Figure 4e, (**c**) laser curve, (**d**) region of interest (ROI), and (**e**) laser curve fitting.

As described above, weld joint identification is performed in L2, and it is the basis of L3 and L4. In the real weld site, the weld joint type generally does not change during one-time tracking; thus, the weld joint type needs to be recognized only at the initial stage L2. In this paper, we consider the recognition of only 40 weld joint images that were captured by the camera in the first second of the welding process. There is no spatter in the joint images at this time. Therefore, we only need to consider the reflection and scattering from the weld surface. This simplification can effectively reduce the difficulty of noise processing and improve the response speed of the system. In this paper, a hybrid filtering algorithm that consists of median filtering with a 5 × 5 square template and an open operation with a 3 × 7 rectangular template is used to remove noise from the weld joint images. Figure 8a,b show the results of image noise processing of Figure 4e. Subsequently, we perform a threshold binary operation to the noise with a grey value below 220. The laser stripe forms clusters in the image and the weld joint positions in each frame are different. Therefore, we extract the laser curve in the image to determine the position of the laser stripe based on the grey centroid method in this paper. This process can be expressed, as follows:

(3) $$\left\{ \begin{array}{l} p_{i}(x)=\sum_{h=j}^{k}\frac{g(h,i)\times h}{\sum_{h=j}^{k}g(h,i)}\\ p_{i}(y)=i \end{array}\right.\;\left(1\leq i\leq 480\right)$$

where *i* is the *i*th row in the image, *p_i_*(*x*) and *p_i_*(*y*) are the coordinates of the points on the laser curve in the image, *g* is the grey value of the current point on the laser stripe, whose value is 0 or 255, *h* is the X coordinate of the current point on the laser stripe, and *k* and *j* are the X coordinates of the left and right edge points, respectively, of the laser stripe in each row of pixels. Figure 8c shows the laser curve extraction results.

#### 3.1.2. Extraction of the ROI

In this paper, the dimensions of the original weld joint images are 640 × 480; thus, processing the whole weld joint image would incur a high computational cost. In fact, the weld joint feature points are only located in the laser curve in the image, and the feature vector is related to the deformation of the laser curve at the weld joint. Therefore, we can only consider the region near the laser curve in the image. For this purpose, we define an ROI near the laser curve in the image to reduce the required number of pixel computations. Moreover, defining an ROI can effectively reduce the probability of noise in the image, thus reducing the difficulty and improving the accuracy of weld joint extraction.

We define a rectangular ROI based on the location of laser curve in the image, and the parameters of the ROI can be determined, as follows:

(4) $$\left\{ \begin{array}{l} X=\min(p_{i}(x))-w_{1}\\ Y=\min(p_{i}(y))\\ W=\max(p_{i}(x))-\min(p_{i}(x))+w_{2}\\ H=\max(p_{i}(y))-\min(p_{i}(y)) \end{array}\right.\;\left(1\leq i\leq M\right)$$

where *X* and *Y* represent the vertex of the top left corner of the ROI, *W* is the width of the ROI, *H* is the length of the ROI, and *w*_1_ and *w*_2_ are the reserved values of the rectangular ROI in the *X* direction. In this paper, we set *w*_1_ to 15 and *w*_2_ to 15 based on a large number of experiments. Figure 8d shows the extraction results.

#### 3.1.3. Laser Curve Fitting

During image preprocessing, the laser curve in the image might be interrupted, as shown in Figure 8d. Therefore, to ensure the accuracy of the subsequent feature vector extraction and fitting of the laser curve in the image, in this paper, the laser curve in the image is fitted by using Equation (5) to calculate the slope:

(5) $$S_{i}=\frac{m_{i}(y)-n_{i}(y)}{m_{i}(x)-n_{i}(x)}\;\left(i\geq 1\right)$$

where *S_i_* represents the slope of the *i*th fit line and *m_i_* and *n_i_* denote the endpoints of the interrupted laser curve in the image. The disconnected curves can be determined by row scanning. Figure 8e shows the line fitting results.

### 3.2. Extraction of Feature Vector

Section II describes the line fitting results. We already know that the weld joint type can be determined based on the abrupt differences of the laser curve at the weld joint in the image. When considering the image characteristics, we propose a method using the laser curve distribution in the image and the weld joint feature point intervals as the basis of a characteristic description to recognize different weld joints types.

#### 3.2.1. Slope Distribution of Laser Curve

**Figure 9:**
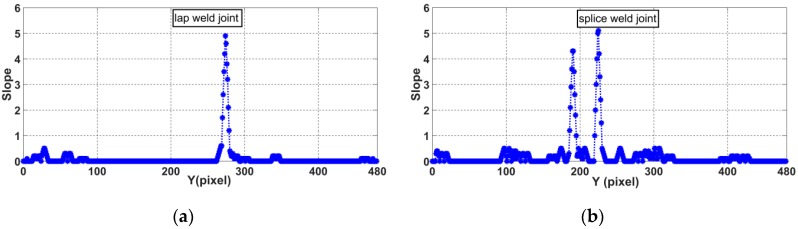
Slope distribution along the laser curve: (**a**) lap weld joint and (**b**) splice weld joint.

At a weld joint, the laser curve will exhibit deformation, which will result in changes in the slope of laser curve. Additionally, the slope distributions of the laser curve that are observed in images of different types of weld joints are different. Therefore, the slope distribution of the laser curve in the image can be used as an effective basis for weld joint type recognition. We define the slope of a point at the laser curve as:

(6) $$\begin{array}{lll} & \left|\frac{p_{i+1}(x)-p_{i-1}(x)}{2}\right|+\left|\frac{p_{i+2}(x)-p_{i-2}(x)}{2}\right|+\left|\frac{p_{i+3}(x)-p_{i-3}(x)}{2}\right|+ & \\ K_{i}= & \frac{\;\;\;\;\;\left|\frac{p_{i+4}(x)-p_{i-4}(x)}{2}\right|+\left|\frac{p_{i+5}(x)-p_{i-5}(x)}{2}\right|\;\;\;\;\;}{5} & \left(5<i<475\right) \end{array}$$

where *K_i_* represents the slope at point *p_i_* on the laser curve in the image and *p_i_*_−1_ to *p_i_*_+5_ represent the adjacent points of *p_i_*. The slopes at the laser curve for each joint type are mainly distributed in the range from 0–5. Figure 9a,b show the lap weld joint image and splice weld joint. For statistical convenience, we define the slope distribution, as follows:

(7) $$\left\{ \begin{array}{ll} K_{i}=1\; & \left(0.6<K_{i}\leq 1.5\right)\\ K_{i}=2\; & \left(1.5<K_{i}\leq 2.5\right)\\ K_{i}=3\; & \left(2.5<K_{i}\leq 3.5\right)\\ K_{i}=4\; & \left(3.5<K_{i}\leq 4.5\right)\\ K_{i}=5\; & \left(4.5<K_{i}\right) \end{array}\right.$$

**Figure 10:**
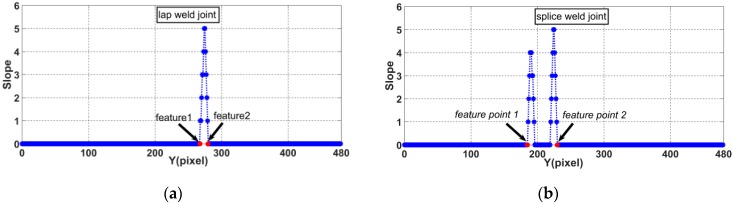
Final slope distribution along the laser curve: (**a**) lap weld joint and (**b**) splice weld joint.

Figure 10a,b show the final laser curves slope distributions of the lap weld joint and splice weld joint images. On this basis, the laser curve slope feature can be defined as Equation (8).

(8) $$\left\{ \begin{array}{l} V_{1}=num\left(K_{i}=1\right)\\ V_{2}=num\left(K_{i}=2\right)\\ V_{3}=num\left(K_{i}=3\right)\\ V_{4}=num\left(K_{i}=4\right)\\ V_{5}=num\left(K_{i}=5\right) \end{array}\right.$$

where *num*() denotes a counting function used to represent the statistics of the slope distribution of laser curve.

#### 3.2.2. Interval of Weld Joint Feature Points

The laser curve slope distribution can be used as an important feature for recognizing the weld joint type; however, there are some weld joints that are very different in appearance, but have similar slope distributions, such as fillet weld joints and v-type weld joints. Therefore, it is necessary to select a feature as a condition to identify the type of weld.

**Figure 11:**
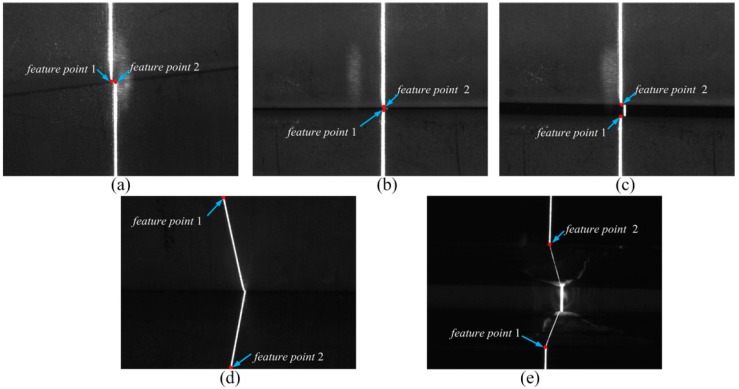
Feature points of weld joints: (**a**) lap weld joint, (**b**) butt weld joint, (**c**) splice weld joint, (**d**) fillet weld joint, and (**e**) v-type weld joint.

The slope of the laser curve will jump twice in the weld joint image. As shown in Figure 10, the positions of the two jumps are marked as *feature point* 1 and *feature point* 2. There is a position difference between *feature point* 1 and *feature point* 2 in the *X* and *Y* directions due to the differences in relative position between the two welding work-pieces. We define *dis_x_* as the pixel difference in the *X* direction and *dis_y_* as the pixel difference in the *Y* direction, as shown in Figure 11. These distances can be calculated as

(9) $$\left\{ \begin{array}{l} V_{6}=dis_{x}=\;|feature\;point\;1_{x}-feature\;point\;2_{x}|\\ V_{7}=dis_{y}=\;|feature\;point\;1_{y}-feature\;point\;2_{y}| \end{array}\right.$$

where *feature point* 1*_x_*, *feature point* 1*_y_*, *feature point* 2*_x_*, and *feature point* 2*_y_* represent the *X* and *Y* coordinates of *feature point* 1 and *feature point* 2, respectively, in the pixel coordinate system.

As shown in Figure 11, the slope of the laser curve changes only at the edge of the weld joint. Therefore, in this paper, *feature point* 1 is defined as the first pixel point at which the slope is greater than 1 and *feature point* 2 is defined as the last pixel point at which the slope is greater than 1, as follows:

(10) $$\left\{ \begin{array}{l} \left\{ \begin{array}{l} feature\;point1=\underset{first}{p_{i}}\\ if:\;abs\left(slope\left(p_{i}\right)-slope\left(p_{i+1}\right)\right)>=1 \end{array}\right.\\ \left\{ \begin{array}{l} feature\;point2=\underset{last}{p_{i}}\\ if:\;abs\left(slope\left(p_{i}\right)-slope\left(p_{i+1}\right)\right)>=1 \end{array}\right. \end{array}\right.$$

**Figure 12:**
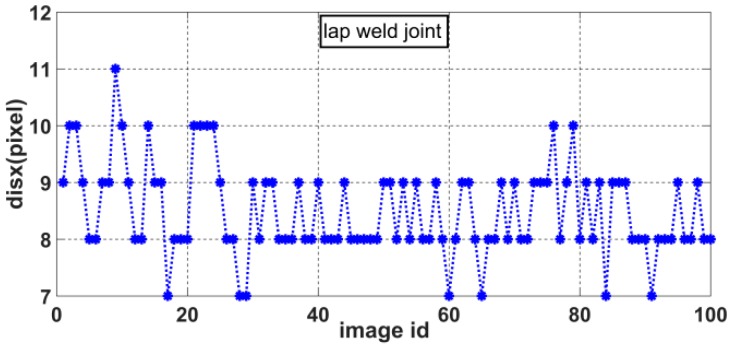
Distribution of *dis_x_* for lap weld joints.

**Figure 13:**
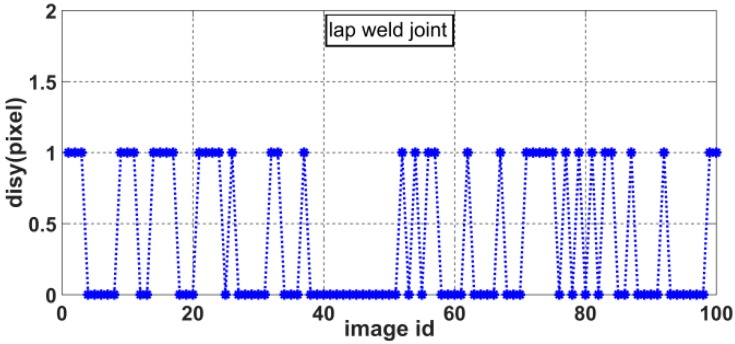
Distribution of *dis_y_* for lap weld joints.

**Figure 14:**
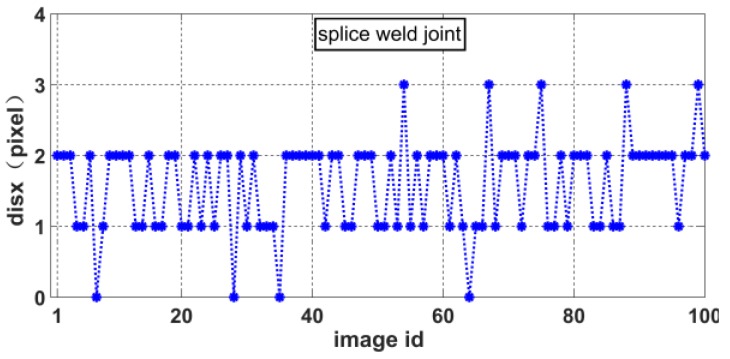
Distribution of *dis_x_* for splice weld joints.

**Figure 15:**
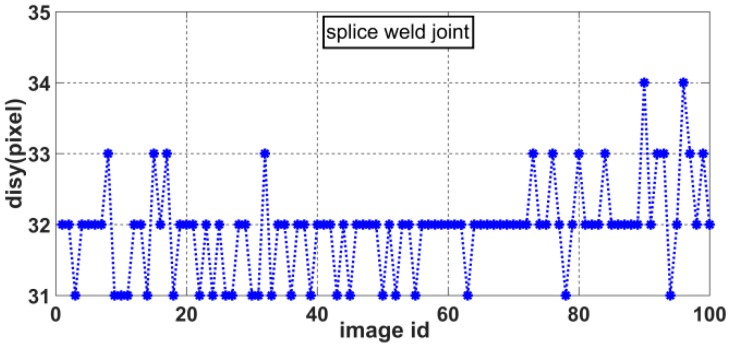
Distribution of *dis_y_* for splice weld joints.

Accordingly, we can obtain the distributions of *disx* and *disy* for various weld joint images. Figure 12, Figure 13, Figure 14 and Figure 15 show the *disx* and *disy* of the lap weld joint and splice weld joint.

On this basis, the feature vector can be expressed as $V=[V_{1},V_{2},V_{3},V_{4},V_{5},V_{6},V_{7}]$.

## 4. Weld Joint Type Recognition Using SVM

The feature vector has been extracted in Section III. Now, a weld joint recognition model should be constructed. Therefore, in this paper, an SVM is adopted as the basis of the recognition model. An SVM is a maximum interval classifier for binary classification that determines the decision plane *f*(*x*) = ***w***^T^***x*** + *b* based on the maximum distance between the plane and some edge data, which are also support vectors.

Suppose that there are two types of data, *A* and *B*, which are labelled with *y* values of {1, −1}, respectively. Afterwards, the decision plane is defined, as follows:

(11) $$\left\{ \begin{array}{l} \mathrm{if}(w^{T}x+b>0)\;\;\mathrm{then}\;(\mathrm{label}:\;1)\\ \mathrm{if}(w^{T}x+b<0)\;\;\mathrm{then}\;(\mathrm{label}:\;-1) \end{array}\right.$$

One of the common methods of solving for $f(x)=w^{T}x+b$ is:

(12) $$\left\{ \begin{array}{l} \min\frac{1}{2}{\left\|w\right\|}^{2}\\ y_{i}\left(w^{T}x+b\right)\geq 1,i=1,\ldots \ldots ,n \end{array}\right.$$

Soft intervals can be introduced to prevent over-fitting, which allows the SVM to make mistakes with some samples and allows some samples to fail to satisfy ***y_i_***(***w*^T^*x*** + ***b***) ≥ 1. However, the number of samples that fail to satisfy the constraint should be as small as possible. Therefore, the relaxation variable *ξ* is introduced, and Equation (12) can be written as:

(13) $$\left\{ \begin{array}{l} \min\frac{1}{2}{\left\|w\right\|}^{2}+C\sum_{i=1}^{n}\xi_{i}\\ \xi_{i}\geq 0\\ y_{i}\left(w^{T}x_{i}+b\right)\geq 1-\xi_{i},i=1,\ldots \ldots ,n \end{array}\right.$$

In the case of linearly inseparable data, it is difficult to find a suitable hyperplane for classifying the two types of data. The common solution to this problem that is adopted in the SVM algorithm is to find a mapping function for mapping the data into higher dimensions; thus, data that are inseparable in lower dimensions can become separable in a higher dimensional space, such that an appropriate hyperplane can be found to classify them. A kernel function ***ĸ***(***x****_i_*, ***x****_j_*) is introduced to map two vectors from the original space into the higher-dimensional space to solve the problem of high-dimensional data and to calculate the inner product during optimization. The input to this kernel function consists of two vectors, and its output is the inner product of the two vectors mapped into the higher-dimensional space. Common kernel functions include linear kernels, polynomial kernels, Gaussian kernels, Laplace kernels, and sigmoid kernels. In this paper, a Gaussian kernel (radial basis function, RBF) is adopted:

(14) $$\kappa \left(x_{i},x_{j}\right)=\exp\left(-g{\left\|x_{i}-x_{j}\right\|}^{2}\right)$$

where *g* is the bandwidth of the Gaussian kernel function. As *g* goes toward infinity, almost all of the samples become support vectors, which can lead to overfitting, as all training samples will be accurately classified. As *g* goes toward zero, the discriminant function of the SVM becomes a constant function, and its classification ability for new samples becomes 0; in other words, it will classify all the samples into the same class, which leads to under-fitting. Therefore, choosing an appropriate bandwidth has great influence on the performance of the model, and it is necessary to find a balance between correctly dividing the current data and ensuring suitability for a wider range of data to ensure that the model has good practical value.

SVMs are mainly used for binary classification; however, there are two main schemes that can be used to adapt them for multi-class classification. The first is the one-to-many scheme, in which multiple classifications are performed, each for identifying whether the samples belong to one particular category. Therefore, for K classes of samples, it is necessary to construct K SVMs. Classification is achieved by comparing the distances between each of the input samples and the hyperplane in each SVM. The second scheme is the one-to-one scheme, which is based on SVMs for distinguishing between two particular classes of samples. Therefore, ***ĸ***(***ĸ*** − 1)/2 SVMs are needed for the K sample classes. The category to which each sample belongs is determined by voting to make the classification decision. The samples are input into all SVMs, and the results of the individual decisions are then counted. The category with the most votes for a given sample is determined to be the category of that sample. In this paper, a total of 10 SVMs are designed for classifying five types of weld joints while using the second scheme.

The selection of the parameter set plays an important role in determining the quality of an SVM model. Additionally, multi-type weld joint recognition involves the selection of parameters for multiple SVM models. In general, there are two methods that can be applied for choosing parameters:

(1) The parameter set for each SVM model (i.e., the model for distinguishing between each pair of weld types) is independently selected, and each model will have its own parameter settings. For example, with this approach, there will eventually be 10 sets of parameters since there are 10 SVM models for the problem considered in this paper.
(2) All of the models share one set of parameters, and the parameters that yield the highest overall performance are selected.

Each method has its advantages. A single parameter set might not be appropriate for all ***ĸ***(***ĸ*** − 1)/2 models. However, the overall accuracy is the ultimate consideration, and individual sets of model parameters may lead to over-fitting of the overall model. Therefore, the second strategy is adopted in this paper, the same set of parameters is used for all of the models, and the parameters are set based on the overall performance.

**Figure 16:**
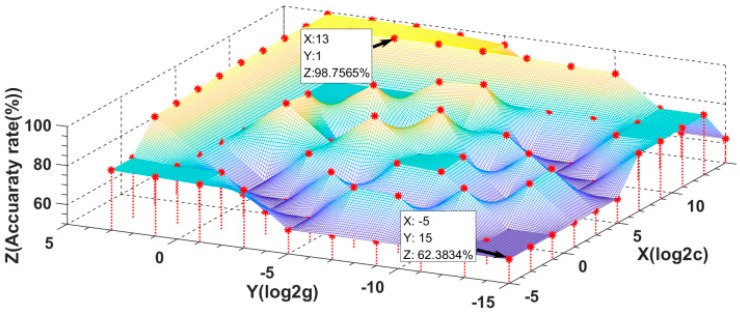
Cross-validation results for the model parameters.

To optimize the parameters, the feature vectors were first normalized to facilitate fast convergence. Subsequently, the parameter set was selected by using the grid search method that is based on five-fold cross-validation. The training samples were divided into five groups. Four groups were treated as the training data each time, and the remaining group was used as the test data. Afterwards, the average five-fold cross-validation accuracy was obtained by averaging the results that were obtained in this way for each set of parameters. Figure 16 shows the results that were obtained for 110 sets of parameters via the cross-validation approach. These results show that when log2C is equal to −5 and log2g is equal to −15, i.e., C = 0.03125 and g = 0.00003, the accuracy that is determined via cross-validation is the lowest, with a value of 62.3834%. When log2C is equal to 13 and log2g is equal to 1, i.e., C = 8192 and g = 2, the accuracy determined via cross-validation is the highest, 98.7565%. Therefore, C = 8192 and g = 2 were used as the parameter settings for model training to establish the weld joint classification model.

## 5. Experimental Results and Analysis

**Figure 17:**
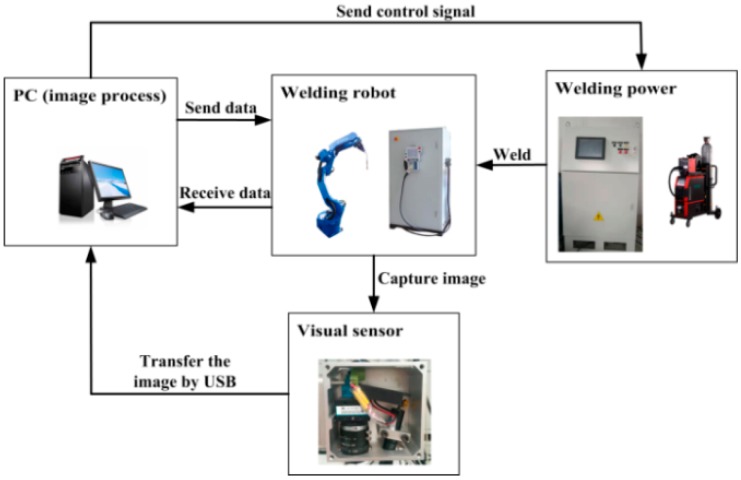
System configuration.

As shown in Figure 17, the welding system that was considered in this paper consisted of five parts: a welding robot, a visual sensor, a robot controller, a computer, and the auxiliary welding equipment.

A visual sensor is installed on the end effector of the robot. For communication, there are cable connections between the auxiliary welding equipment and the robot controller, between the computer and the robot controller, and between the robot controller and the welding robot, and the visual sensor transmits image data to the computer through a USB connection.

The five weld joint types that were considered for recognition in this paper were lap weld joints, butt weld joints, splice weld joints, fillet weld joints, and v-type weld joints. Table 2 shows the parameters of each weld type.

### 5.1. Experimrntal Results

For this analysis, 600 weld joint images, including 120 images for each type of joint, were selected as the training images. A total of 250 weld joint images were selected for testing: images 1–50 were images of lap weld joints, labelled with a value of 1; images 51–100 were the images of butt weld joints, labelled as 2; images 101–150 were images of splice weld joints, labelled as 3; images 151–200 were images of fillet weld joints, labelled as 4; and, images 201–250 were images of v-type weld joints, labelled as 5. All of the experiments that were reported in this paper were performed on a computer with an Intel i5-6500 CPU, with a main frequency of 3.2 GHz and 8 GB of RAM.

**Figure 18:**
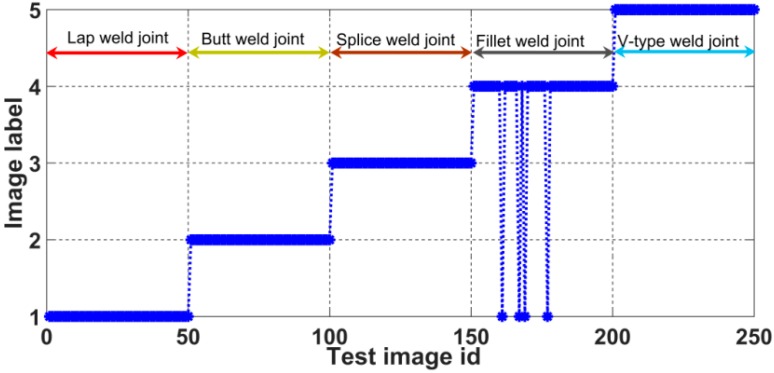
Results of weld joint identification using the proposed method.

According to the final experimental results, the recognition accuracy achieved 98.4%, and the computational cost of single-image recognition is 148.23 ms. Figure 18 shows the recognition results for various weld joint images.

### 5.2. Comparison of Weld Joint Image Feature Extraction Methods

**Figure 19:**
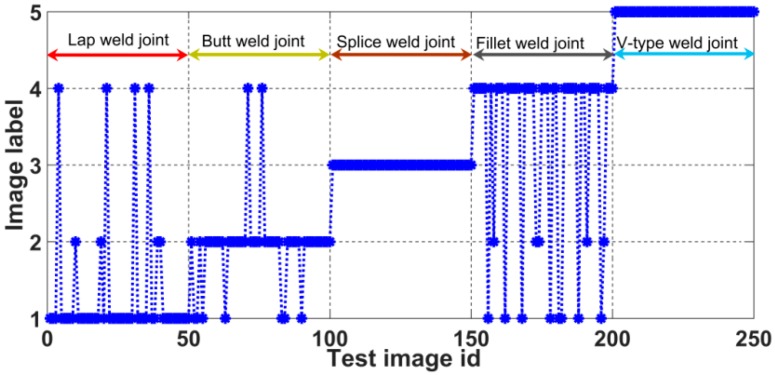
Results of weld joint identification using the reference [23].

We compared the proposed feature extraction algorithm for weld images with the weld image feature extraction method presented in reference [23] to verify the effectiveness and superiority of the feature extraction method proposed in this paper. The cited paper presents a comparison with previously proposed weld joint feature extraction algorithms and it shows that the selected method achieves better recognition accuracy with a lower computational cost; thus, it can be used as an object of comparison in this paper. We used both feature extraction algorithms to extract feature vectors with which to build SVM models. Table 3 shows the final recognition accuracies and computational costs of the two methods. Figure 19 shows the results of weld image recognition.

A comparison reveals that the recognition accuracy and computational cost of the weld image feature extraction algorithm that was proposed in this paper are better than those of the feature extraction method presented in reference [23]. The vertical distance from the groove to the surface of the weld joint is used to construct the feature vector. This feature vector is relatively simple. Consequently, this method is more suitable for weld joint types or weld grooves that exhibit large differences. Weld joints that do not show significant groove differences will be difficult to distinguish. The method of reference [23] misidentifies some lap weld joints, splice weld joints, and fillet weld joints, because the weld grooves of these three types of joints are relatively small, which makes it difficult to differentiate among them, as shown in Figure 19. By contrast, the method that was proposed in this paper not only utilized the characteristics of the weld joint, but also accounted for laser deformation, which made it suitable for a wider scope of applications and enabled it to achieve a higher accuracy rate.

### 5.3. Comparision of Classification Methods

Currently, the most commonly applied classification algorithms include logistic regression, the K-nearest- neighbour algorithm, the decision tree algorithm, Bayesian classification, the SVM algorithm, and neural networks. The SVM results were compared with the results of other classification methods to verify the effectiveness and applicability of the SVM method selected in this paper. Logistic regression is mainly used for binary classification, since the number of weld types considered here is greater than two, logistic regression is not a suitable classification algorithm for the problem of interest. Bayesian classification is based on the premise that the posterior probability can be obtained with the prior probability. Bayesian classification is not suitable for the problem of interest because it is difficult to find a suitable prior probability for the problem considered in this paper. Neural networks have high requirements in terms of the number of samples needed and they are not suitable for classification with small sample sizes, and they require a high performance GPU for training and testing; they are not suitable for welding industrial sites. Therefore, the SVM model that was established in this paper was compared with the K-nearest-neighbour algorithm and the decision tree algorithm. The classification accuracy of each algorithm and the computational cost of single image recognition were analysed based on the feature vectors that were extracted in this paper.

The K-nearest-neighbour algorithm recognizes the weld joint type by calculating the Euclidean distances between the feature vector of the weld joint to be recognized and the feature vectors of all known joint images. In the decision tree algorithm, a decision tree is recursively generated by selecting features as criteria for node splitting. The traditional ID3 algorithm was used in this experiment. Table 4 shows the final experimental results.

The experimental results show that the joint recognition system that is based on the proposed SVM model is superior to the K-nearest neighbour algorithm in terms of both recognition accuracy and computational cost. By contrast, the time cost of the decision tree algorithm is lower than that of the SVM algorithm by 9.88 ms; however, its accuracy rate is only 90.3%. Therefore, based on comprehensive consideration of the computational cost and accuracy, we conclude that the SVM model that is presented in this paper is also superior to the decision tree algorithm.

### 5.4. Robustness Testing of the Proposed Weld Joint Recognition Method

**Figure 20:**
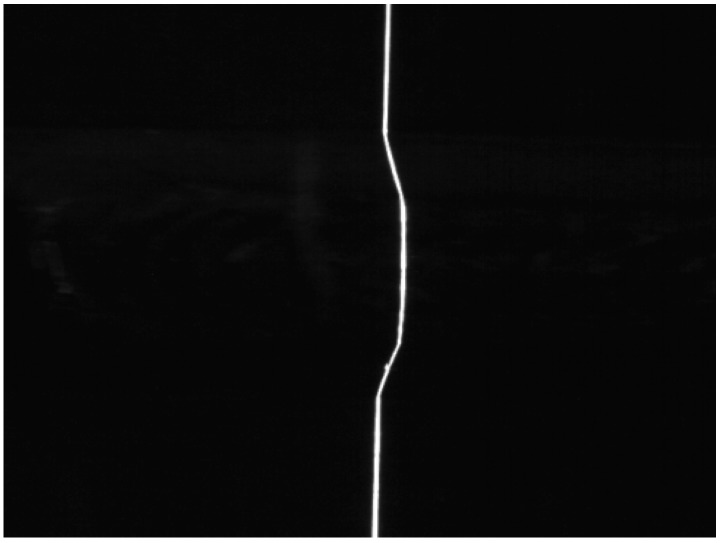
Laser curve image of a filler layer weld joint.

We added a new weld joint type to the model to verify the robustness of the proposed weld recognition algorithm. The new weld joint type is “filler layer weld joint”. Figure 20 shows a laser curve image of such a filler layer weld joint. During the actual welding process, the welding voltage and current are lower than those in the bottom layer, because one layer has already been welded, and the swing range of the welding torch should be smaller. Thus, the weld characteristics meet the needs of weld joint recognition, as presented in this paper. With this addition of this weld joint type, the final recognition accuracy of the new weld joint recognition system is 98.1%. When compared with the previous performance, the accuracy of the proposed system is reduced by only 0.3%, thus showing that the proposed joint recognition system exhibits good robustness.

It is necessary to test the recognition results for different weld sizes to verify the generalizability of the proposed recognition algorithm. Therefore, along with the additional weld joint mentioned above, our system was applied to weld joint image of different sizes, as shown in Table 5. Ultimately, the recognition accuracy of the new model is 98.4% and the computational cost is 148.23 ms. In fact, the changes in weld size have no effect on the recognition accuracy.

**Figure 21:**
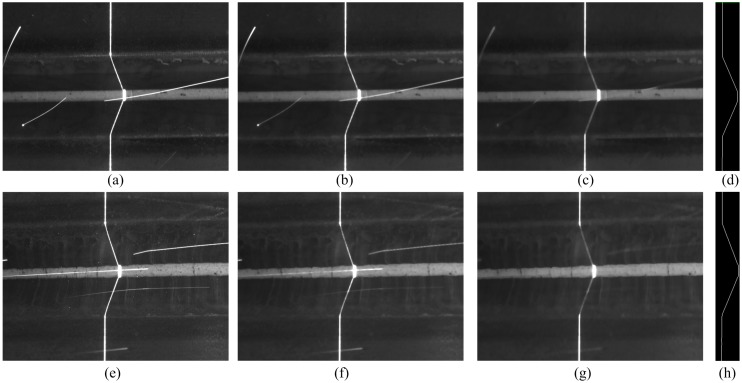
(**a**,**e**) the original image with splash and arc light, (**b**,**f**) the image after median filter, (**c**,**g**) The image after open operation, and (**d**,**h**) the process result.

In this study, we recognize the welding joint type before welding begins. However, there might be multiple welding robots working together in an actual welding workshop. In this case, the other welding robots affects the welding joint image, and the arc light and splash generated by other welding robots appear in the image. We input the image with splash and arc light into our welding system to test the effectiveness of the proposed algorithm in the case of external noise interference. The results show that the proposed algorithm can effectively process an image with splash and arc light, as shown in Figure 21.

## 6. Conclusions

In this paper, we proposed an algorithm to address the low adaptability and automation of traditional weld joint feature extraction algorithms that are based on visual tracking sensors for determining welding parameter configurations in multi weld joint type environments. Based on images that were captured by a visual tracking sensor, a weld joint type recognition algorithm that considers the slope distribution at points along the laser curve and the distance between feature points of a weld joint to construct the feature vector for SVM classification is proposed. The following conclusions can be drawn from the experimental results:

(1) The proposed weld joint recognition system can accurately identify various weld joint types.
(2) An image feature extraction method is proposed to extract two kinds of feature information, which can increase the recognition information and improve the recognition accuracy.
(3) The weld joint image feature extraction algorithm that was proposed in this paper offers better recognition accuracy and a lower computational cost than algorithms from other papers.
(4) The weld joint recognition system that was proposed in this paper exhibits good robustness. After the addition of a new weld joint type, this method can still effectively recognize different types of joints with higher recognition accuracy.
(5) In future work, we will attempt to achieve online recognition to allow for weld joint types to be recognized during the actual welding process, which will require a stronger ability to deal with noise.

## Figures and Tables

**Table 1:** Camera parameters.

Parameter	Value
Resolution	640 × 480 (pixel)
Frame rate	40 fps
Sensor type	CMOS
Pixel size	6 μm × 6 μm

**Table 2:** Weld joint parameters.

Type of Weld Joint	Dimensions of Workpieces Plates
Lap weld joint	600 mm × 150 mm × 3 mm
Butt weld joint	600 mm × 150 mm × 3 mm
Splice weld joint	600 mm × 150 mm × 3 mm
Fillet weld joint	600 mm × 150 mm × 3 mm
V-type weld joint	800 mm × 150 mm × 20 mm

**Table 3:** Recognition results obtained with different feature extraction algorithms.

Feature Extraction Algorithm	Accuracy Rate	Computational Cost
This paper	98.4%	148.23 ms
Reference [23]	89.2%	165.62 ms

**Table 4:** Comparison of recognition results.

Classification Algorithm	Accuracy Rate	Computational Cost
SVM	98.40%	148.23 ms
K-nearest-neighbour	97.00%	984.92 ms
Decision tree	90.30%	138.35 ms

**Table 5:** New weld joint parameters.

Type of Weld Joint	Dimensions of Work-Pieces Plates
Lap weld joint	600 mm × 150 mm × 5 mm
Butt weld joint	600 mm × 150 mm × 5 mm
Splice weld joint	600 mm × 150 mm × 5 mm
Fillet weld joint	600 mm × 150 mm × 5 mm
V-type weld joint	800 mm × 150 mm × 25 mm

## References

[1] Chen S.B., Lv N. (2014). Research evolution on intelligentized technologies for arc welding process. J. Manuf. Process..

[2] Wang Z. (2015). An imaging and measurement system for robust reconstruction of weld pool during arc welding. IEEE Trans. Ind. Electron..

[3] You D., Gao X., Katayama S. (2015). WPD-PCA-based laser welding process monitoring and defects diagnosis by using FNN and SVM. IEEE Trans. Ind. Electron..

[4] Maiolino P., Woolley R., Branson D., Benardos P., Popov A., Ratchev S. (2017). Flexible robot sealant dispensing cell using RGB-D sensor and off-line programming. Robot. Comput. Integr. Manuf..

[5] Zhang Z.F., Chen S.B. (2017). Real-time seam penetration identification in arc welding based on fusion of sound, voltage and spectrum signals. J. Intell. Manuf..

[6] Xu Y.L., Zhong J.Y., Ding M.Y., Chen S.B. (2013). The acquisition and processing of real-time information for height tracking of robotic GTAW process by arc sensor. Int. J. Adv. Manuf. Technol..

[7] Lü X.Q., Gu D.X., Wang Y.D., Qu Y., Qin C., Huang F.Z. (2018). Feature extraction of welding seam image based on laser vision. IEEE Sens. J..

[8] Li X.H., Li X.D., Khyam M.O., Ge S.S. (2017). Robust welding seam tracking and recognition. IEEE Sens. J..

[9] Rodríguez-Martín M., Rodríguez-Gonzálvez P., González-Aguilera D., Fernández-Hernández A.J. (2017). Feasibility study of a structured light system applied to welding inspection based on articulated coordinate measure machine data. IEEE Sens. J..

[10] Fang Z.J., Xu D., Tan M. (2013). Vision-based initial weld point positioning using the geometric relationship between two seams. Int. J. Adv. Manuf. Technol..

[11] Kong M., Shi F.H., Chen S.B., Lin T. (2007). Recognition of the initial position of weld based on the corner detection for welding robot in global environment. Robotic Welding.

[12] Zhu Z.Y., Lin T., Piao Y.J., Chen S.B. (2005). Recognition of the initial position of weld based on the image pattern match technology for welding robot. Int. J. Adv. Manuf. Technol..

[13] Zhou L., Lin T., Chen S.B. (2006). Autonomous acquisition of seam coordinates for arc welding robot based on visual servoing. J. Intell. Robot. Syst..

[14] Dinham M., Fang G. (2013). Autonomous weld joint identification and localisation using eye-in-hand stereo vision for robotic arc welding. Robot. Comput. Integr. Manuf..

[15] Sung K., Lee H., Choi Y.S., Rhee S. (2009). Development of a multi-line laser vision sensor for joint tracking in welding. Weld. J..

[16] Lee J.P., Wu Q.Q., Park M.H., Park C.K., Kim I.S. (2014). A study on optimal algorithms to find joint tracking in GMA welding. Int. J. Eng. Sci. Innov. Technol..

[17] Fang J.F., Jing F.S., Yang L. (2019). A precise seam tracking method for narrow butt seams based on structured light vision sensor. Opt. Laser Technol..

[18] Zou Y.B., Chen T. (2018). Laser vision seam tracking system based on image processing and continuous convolution operator tracker. Opt. Lasers Eng..

[19] Fang Z., Xu D. Image-based visual seam tracking system for fillet joint. Proceedings of the 2009 IEEE International Conference on Robotics and Biomimetics (ROBIO).

[20] Li X.D., Li X.H., Ge S.Z. (2017). Automatic welding seam tracking and identification. IEEE Trans. Ind. Electron..

[21] Qian B.F., Liu N.S., Liu M.Y., Lin H.L. (2007). Automatic recognition to the type of weld seam by visual sensor with structured light. Nanchang Univ. Eng. Technol..

[22] Li Y., Xu D., Tan M. (2006). Welding joints recognition based on Hausdorff distance. Chin. High Technol. Lett..

[23] Fan J.F., Jing F.S., Fang Z.J. (2017). Automatic recognition system of welding seam type based on SVM method. Int. J. Adv. Manuf. Technol..

[24] Zeng J., Cao G.Z., Li W.B., Chen B.C. An algorithm of hand-eye calibration for arc welding robot. Proceedings of the 2019 16th International Conference on Ubiquitous Robots (UR).

[25] Zhang Z. (2000). A flexible new technique for camera calibration. IEEE Trans. Pattern Anal. Mach. Intell..

[26] Li W.B., Cao G.Z., Sun J.D., Liang Y., Huang S.D. A calibration algorithm of the structured light vision for the arc welding robot. Proceedings of the 2017 14th International Conference on Ubiquitous Robots and Ambient Intelligence (URAI).

